# Evaluation of a Recombinant *Flavobacterium columnare* DnaK Protein Vaccine as a Means of Protection Against Columnaris Disease in Channel Catfish (*Ictalurus punctatus*)

**DOI:** 10.3389/fimmu.2019.01175

**Published:** 2019-06-06

**Authors:** Miles D. Lange, Jason Abernathy, Bradley D. Farmer

**Affiliations:** Harry K. Dupree Stuttgart National Aquaculture Research Center, Agricultural Research Service, United States Department of Agriculture, Stuttgart, AR, United States

**Keywords:** recombinant protein, bath immersion, *Flavobacterium columnare*, channel catfish, antibody response, RNA sequencing

## Abstract

*Flavobacterium columnare* causes substantial losses among cultured finfish species. The Gram-negative bacterium is an opportunistic pathogen that manifests as biofilms on the host's mucosal surfaces as the disease progresses. We previously demonstrated that the dominant mucosal IgM antibody response to *F. columnare* is to the chaperone protein DnaK that is found in the extracellular fraction. To establish the efficacy of using recombinant protein technology to develop a new vaccine against columnaris disease, we are reporting on two consecutive years of vaccine trials using a recombinant *F. columnare* DnaK protein (rDnaK). In year one, three groups of channel catfish (*n* = 300) were immunized by bath immersion with a live attenuated *F. columnare* isolate, rDnaK or sham immunized. After 6 weeks, an *F. columnare* laboratory challenge showed a significant increase in survival (>30%) in both the live attenuated and rDnaK vaccines when compared to the non-immunized control. A rDnaK-specific ELISA revealed significant levels of mucosal IgM antibodies in the skin of catfish immunized with rDnaK at 4- and 6-weeks post immunization. In the second year, three groups of channel catfish (*n* = 300) were bath immunized with rDnaK alone or with rDnaK after a brief osmotic shock or sham immunized. After 6 weeks a laboratory challenge with *F. columnare* was conducted and showed a significant increase in survival in the rDnaK (> 25%) and in rDnaK with osmotic shock (>35%) groups when compared to the non-immunized control. The rDnaK-specific ELISA demonstrated significant levels of mucosal IgM antibodies in the skin of catfish groups immunized with rDnaK at 4- and 6-weeks post immunization. To further understand the processes which have conferred immune protection in the rDnaK group, we conducted RNA sequencing of skin samples from the non-immunized (*n* = 6) and rDnaK treated channel catfish at 1-week (*n* = 6) and 6 weeks (*n* = 6) post immunization. Significantly altered gene expression was identified and results will be discussed. Work to further enhance the catfish immune response to *F. columnare* rDnaK is underway as this protein remains a promising candidate for additional optimization and experimental trials in a production setting.

## Introduction

*Flavobacterium columnare*, the causative agent of columnaris disease generates substantial mortality during the production of freshwater farmed finfish species (1, 2). Classically, columnaris disease has been shown to predominantly involve the external mucosal surfaces of fish, which can lead to the rapid and widespread destruction of the gills, skin, and fins. *F. columnare* is ubiquitous in the aquatic environment and outbreaks are often triggered during the spring and summer months of the production cycle (3, 4). Intensive rearing of food fish is well-suited for the transmission of *F. columnare* and in these settings the pathogen is opportunistic and outbreaks are common, as fish experience stressors including increased rearing density, unnecessary handling and poor water quality (5–7). As food fish production continues to expand, the frequency of columnaris disease will only continue to increase within the aquaculture industry. The regulation of treatments and resistance to available antibiotics means that alternative methods of disease protection will be required (8).

Many vaccines have been developed and used in the aquaculture industry to prevent expensive losses which occur throughout the production cycle due to a wide variety of infectious diseases (9–12). Some of the earliest fish vaccine preparations were isolated bacterial pathogens that were cultured, killed, and then used to immunize fish to examine their overall immunogenicity (13–16). As we began to understand the nature of adaptive immunity in teleost fish, killed bacterins gave way to live-attenuated bacterial vaccines engineered to cause little or no disease and offer more potential for stimulating the adaptive immune response (17, 18).

Channel catfish has served for many years as a good model for examining teleost immune function (19–22). Studies to evaluate different tissue-derived catfish transcriptomes under different conditions during laboratory challenges have allowed for new insight into the pathogenesis of different Gram-negative bacteria (23–25). Recently our lab characterized the mucosal IgM antibody response in channel catfish to an iron-attenuated *F. columnare* isolate after bath immunization (26). We observed that the DnaK protein was primarily found in the extracellular fraction of different *F. columnare* isolates and was predominantly reactive with mucosal IgM antibodies. The DnaK protein has also been detected in the extracellular fraction of the fish pathogen, *Aeromonas salmonicida*, where the authors classified it as a moonlighting protein and pondered on whether it could be a potential vaccine target (27). Heat shock proteins represent a conserved family of proteins and there is evidence to suggest that these proteins are prominent in activating an adaptive immune response in mammals (28–31).

Recent advances in recombinant protein technology have made the production and subsequent testing of individual immunogens quite effective (32–35). In the current work, we tested the ability of a recombinant *F. columnare* DnaK protein (rDnaK) to induce a catfish mucosal IgM antibody response and to protect against columnaris disease. We also examined molecular mechanisms through which protection may be induced using high-throughput RNA sequencing of a mucosal tissue through multiple weeks post-immunization. These combined results suggest that the *F. columnare* rDnaK protein is a candidate for additional studies to improve and validate these experimental trials in a commercial environment.

## Materials and Methods

### Bacteriology and Extracellular Protein Preparation

*F. columnare* isolate LV-359-01 was retrieved from frozen glycerol stocks stored at −80°C and streaked onto *F. columnare* Growth Medium (FCGM) (36, 37). After 48 h of growth at 28°C, the isolate was dislodged from the agar using a sterile loop and inoculated into 5 mL of FCGM (starter) overnight at 28°C. The next day the 5 mL starter was placed into 1 L of FCGM and incubated at 28°C for 24 h at 200 RPM. To produce a live bacterin, LV-359-01 was also grown under iron-limiting conditions with FCGM that contained 100 μM of the high-affinity iron chelator 2, 2′-bipyridyl (Sigma-Aldrich, St. Louis, MO). The use of 2, 2′-bipyridyl to attenuate virulence for this isolate of *F. columnare* was an observation made from our previous work (38). For the live bacterin (LV-DP), we subcultured LV-359-01 in FCGM broth under iron limiting conditions for 24 h and used it immediately to bath immunize channel catfish for the Year 1 study. To isolate extracellular proteins, bacterial suspensions were centrifuged using an Eppendorf 5810R at 6,320 × *g* for 20 min. The extracellular portion (ECP) was aspirated into a new tube and centrifuged again for an additional 10 min. The ECP was aspirated and concentrated using 3K MWCO Amicon Ultra-15 centrifugal filter units (EMD Millipore, Billerica, MA). All ECP fractions had a 5% (v/v) protease inhibitor cocktail (Sigma Aldrich, St. Louis, MO) added prior to estimating the total protein concentration using the Coomassie Plus assay kit (ThermoFisher Scientific, Waltham, MA) with bovine serum albumin (Sigma Aldrich, St. Louis, MO) as the standard. Absorbance was read at a wavelength of 595 nm with a BioTek Synergy H1 plate reader operating under Gen5 software (Winooski, VT).

### Construction, Expression, and Evaluation of Recombinant *F. columnare* DnaK Protein

To construct the expression vector, we first identified the annotated DnaK protein sequence from the *Flavobacterium columnare* isolate ATCC 49512 genome (AEW86253.1). The full-length nucleotide sequence was submitted for codon optimization, incorporation of restriction sites, *NcoI*, and *XhoI* flanking the coding region, gene synthesis and cloning into pET-28a(+) expression vector with a C-terminal His-tag (Genscript, Piscataway, NJ). During this process the second codon of the DnaK sequence was altered from AGC to GGC for incorporation of the NcoI restriction site. The codon optimized recombinant *F. columnare* DnaK coding sequence which shared 71.4% nucleotide identity (amino acid identity 100% to AEW86253.1 protein sequence) to the *F. columnare* DnaK 49512 genomic clone was verified through Sanger sequencing. The expression vector was transformed into *Escherichia coli* strain BL21 (DE3) (Invitrogen, Carlsbad, CA) on Luria– Bertani (LB) agar supplemented with kanamycin (50 mg/mL) and grown at 37°C. For expression, a recombinant *F. columnare* DnaK clone was retrieved from a frozen glycerol stock stored at −80°C and streaked onto LB agar plate supplemented with kanamycin and grown overnight. A single colony was then cultured overnight in 50 mL of LB broth supplemented with kanamycin. A flask containing 250 mL of fresh LB broth with kanamycin was inoculated with 10 mL of the overnight culture and incubated for 2 h under the same culture conditions. Recombinant protein expression was induced by adding isopropyl thiogalactoside (IPTG) at a 1 mM final concentration and cultured for an additional 2 to 6 h. The rDnaK protein was purified from the *E. coli* pellet under native conditions according to the manufacturer's guidelines (Ni-NTA handbook, Qiagen) using a HisPur Ni-NTA spin column (ThermoFisher Scientific, Waltham, MA). Protein concentration was estimated as described above. Aliquots were dispensed and kept at −20°C until needed. SDS gel electrophoresis was conducted to analyze expressed protein using 10% TGX stain-free gels and buffers of the mini-protean system (Biorad, Hercules, CA). We loaded 5 μg of bacterial lysates or eluted rDnaK protein onto SDS gels with the Precision Plus gel marker (Biorad, Hercules, CA), stained using Simple Blue Safe (ThermoFisher Scientific, Waltham, MA) and visualized using a Biorad ChemiDoc XRS+ gel system operating under Image Lab 3.0 software. The rDnaK protein was excised from the SDS gel and mass spectrometry analysis was conducted as previously described (26).

### Bath Immunizations and *F. columnare* Challenges

All channel catfish fingerlings were reared at the Harry K. Dupree Stuttgart National Aquaculture Research Center in Stuttgart, Arkansas, USA.

In year one, 300 catfish in each of three groups (average weight 5 g) were bath immunized under different conditions prior to being stocked into 200 L tanks that received filtered well water and aeration from submerged air stones. Non-immunized control catfish were sham vaccinated, an iron attenuated LV-359-01 (LV-DP) culture was used to bath immunize catfish (calculated dose of 4 x 10^7^ CFU/mL), and the recombinant *F. columnare* DnaK protein (rDnaK) was used to bath immunize catfish (100 μg/mL). All bath immunizations were done statically for 30 min with aeration. Fish were maintained on pelleted catfish feed daily (35% protein, 2.5% fat; Delta Western, Indianola, Mississippi).

After 6 weeks, the non-immunized control and two immunized groups were challenged with virulent *F. columnare* LV-359-01 with a calculated dose of 8.5 × 10^6^ CFU/mL. For each group there were three challenge replicates, where catfish (*n* = 44) were stocked into 18 L aquaria containing 10 L of well water. Water was provided through the ultra-low-flow water delivery system at a rate of 30 mL/min (5, 39). An additional tank with catfish (*n* = 44) was used as the non-challenged control. The number of mortalities from each group was recorded twice daily over 6 days for survival curves.

In year two, 300 catfish in each of three groups (average weight 5 g) were bath immunized. Control catfish were sham vaccinated, and rDnaK was used to bath immunize catfish (200 μg/mL). For the third group (rDnaK + salt) a bath immunization occurred just after hyper-osmotic induction. Catfish were first immersed in a 4.5% (w/v) NaCl bath for 2 min and then bath immunized with rDnaK (200 μg/mL). All bath immunizations were done statically for 30 min with aeration.

Six weeks after the bath immunizations, the different groups were challenged (using the same system described above) with virulent *F. columnare* LV-359-01 at a calculated dose of 1.0 × 10^6^ CFU/mL. For each group there were three challenge replicates, where catfish (*n* = 40) were stocked into 18 L aquaria containing 10 L of filtered well water. An additional tank with catfish (*n* = 40) was used as the non-challenged control. The number of mortalities from each group was recorded twice daily over 5 days for survival curves. In all studies, temperature and dissolved oxygen were measured using an YSI Pro20 dissolved oxygen meter (Yellow Springs, Ohio). Each fish culture tank received aeration from submerged air stones and all studies were kept at 12 h light: 12 h dark photoperiod.

### Tissue Sampling and ELISAs

In years one and two, catfish from the non-immunized control or immunized groups were sampled at 2, 4, 6, 8, and 10 weeks. In year 2, catfish were also sampled at 1-week post immunization. For tissue sampling, catfish were euthanized in a solution of MS-222 for 10 min (300 mg/L, Syndel USA, Ferndale, WA), and whole blood was collected using 70 μL heparinized capillary tubes (ThermoFisher Scientific, Waltham, MA) from the caudal vein after tail fin removal and then allowed to clot overnight at 4°C. Blood samples were centrifuged at 10,000 × *g* for 10 min using an Eppendorf Minispin; the serum (20–50 μL) was removed and stored at −20°C until needed. After blood collection we proceeded with the preparation of excised skin for tissue culture as described (26). Briefly we wiped down the surface of the skin on both sides with a 70% ethanol solution. Then using sterile instruments, we dissected two consecutive 1.5 mm^2^ skin pieces (between the lateral line and dorsal fin), washed them with Leibovitz's L-15 medium (ThermoFisher Scientific, Waltham, MA), and placed them into 300 μL of complete Leibovitz's L-15 medium (10% FBS, penicillin/streptomycin, amphotericin, gentamicin) in a 48-well plate at 28°C for 24 h. The next day the skin explant tissue culture medium was removed and RNA*later* (ThermoFisher Scientific, Waltham, MA) was added to sufficiently cover the skin explants and stored at −80°C.

We used an indirect ELISA to measure the serum and skin based IgM antibodies as described with some modifications (22, 26). Prior to conducting these ELISA experiments; we established the amount of rDnaK required to bind and generate a consistent OD signal using the DnaK specific mAb 8E2/2 (Enzo Life Sciences, Farmingdale, NY). Pierce 96-well polystyrene plates (ThermoFisher Scientific, Waltham, MA) were coated with 100 μL of 10 μg/mL of *F. columnare* LV-359-01 ECP or 10 μg/mL of rDnaK protein in a sodium bicarbonate buffer. Plates were then rinsed three times with 1x PBS with 0.05% Tween-20 (PBST) and then incubated for 1 h in blocking solution (PBST with 5% milk). One hundred μL of serum (1:100) or skin explant medium (1:4) were further serially diluted to 1:1600 or 1:32 in 1x PBS on the horizontal axis of an antigen-coated ELISA plate and incubated at room temperature for 1 h. Plates were rinsed as above and 100 μL of anti-channel catfish IgM mouse monoclonal 9E1 antibody (40) was added at 1:500 dilution in blocking solution. The anti-trout IgM monoclonal antibody was used as an isotype control (41). After 1 h of incubation at room temperature, plates were washed with PBST and 100 μL of sheep anti-mouse HRP-conjugated IgG (GE Healthcare, Pittsburgh, PA) was diluted 1:5000 in blocking solution and incubated for 30 min at room temperature. Plates were rinsed three times with PBST, and 100 μL of 1-Step Ultra TMB-ELISA substrate solution (Thermo Fisher Scientific, Waltham, MA) was added. The peroxidase reaction was stopped after 20 min with 100 μL of 3M H_2_SO_4_ and assessed at 490 nm with a BioTek Synergy H1 plate reader operating under Gen5 software (Winooski, VT).

Differences among the serum or skin based *F. columnare* ECP or rDnaK specific IgM antibody levels were evaluated using One-Way ANOVA. Survival data was analyzed using Kaplan-Meier log rank survival analysis. Probabilities of *P* < 0.05 were considered statistically significant. All statistical tests were performed using GraphPad Prism version 7.0 (San Jose, California).

### RNA Sequencing of Skin Explants in Year 2

Skin explant tissues from non-immunized catfish (*n* = 6), catfish 1-week after treatment with rDnaK (*n* = 6) and catfish 6 weeks post-treatment with rDnaK (*n* = 6) were randomly selected for transcriptome analyses. Skin explants stored in RNA*later* at −80°C as described were used to create libraries for Illumina (San Diego, CA) high-throughput RNA sequencing (RNAseq). First, total RNA was extracted from each tissue using the Qiagen RNeasy Mini Kit (Germantown, MD) per the manufacturer's recommendation. Total RNA was treated with Amplification Grade DNase I (Sigma-Aldrich, St. Louis, MO) according to the package insert and then precipitated with sodium acetate—ethanol and reconstituted in nuclease-free water. Samples were assessed for quality using the Agilent Bioanalyzer RNA 6000 Nano Kit (Santa Clara, CA), where RNA Integrity Numbers > 8 were considered valid. For each sample, DNase-treated total RNA was standardized to 100 ng by spectrophotometry (Bio-Tek, Winooski, VT) and sequencing libraries were prepared using the NEBNext Ultra II Directional RNA Library Prep Kit for Illumina with the NEBNext Poly(A) mRNA Magnetic Isolation Module (New England Biolabs, Ipswich, MA). Each sample was barcoded using the NEBNext Multiplex Oligos for Illumina, Index Primers Sets 1 and 2 (New England Biolabs). RNAseq libraries were sent to a service provider (Novogene, Sacramento, CA) for 150 bp paired-end sequencing on an Illumina HiSeq X Ten.

### Bioinformatics

De-multiplexed, raw reads were provided by the service provider (Novogene). The Trim_Galore! software was used to remove Illumina adapters and trim low-quality ends from each read at a cut-off score of Q20 (42). Bowtie2 was used to align reads against the channel catfish RefSeq transcriptome from the IpCoco_1.2 genome assembly (43). The output from Bowtie2 was piped into the eXpress software using the parameters as recommended by eXpress when using the Bowtie2 aligner and included directional information from the reads (44). Effective read counts for each sample were then used for statistical comparisons by the DESeq2 package of R-bioconductor to generate lists of Differentially Expressed Genes (DEGs) between each control-treatment group. DEG assignment was significant where *P*-adj < 0.05 and at a fold-change > 1.5. Finally, DEG lists were manually aggregated to the gene-level to provide a final list of candidate genes (45). Raw and processed data along with a complete description of computational methods were submitted to the NCBI Gene Expression Omnibus (GEO) accessible under the accession number GSE121116.

### Functional

Enrichment testing was performed on DEG lists to populate gene ontologies (GO). GO categories include Biological Process (BP), Cellular Component (CC), and Molecular Function (MF). The Blast2GO PRO software was used to perform all functional analyses (46). First, the channel catfish RefSeq transcriptome from the IpCoco_1.2 genome assembly was up-loaded into Blast2GO where sequences underwent BLASTx (E-value< 1e-3), enzyme commission assignment, InterPro protein sequence analyses along with GO term assignment, where available. Then, each DEG list was evaluated for enrichment first using Fisher's exact test at a False Discovery Rate (FDR < 0.05). To do this, the functionally-annotated catfish transcriptome was used as a “reference set” of genes while the DEG list from each comparison was used as the “test set” of genes in the Blast2GO software. For additional functional information, Gene Set Enrichment Analysis (GSEA) was also performed in the Blast2GO software (47). To do this, differential expression between each comparison of interest was re-analyzed in DESeq2, where a shrinkage estimator (coef = 2) was applied. All genes were then ranked by the ratio of significance of expression (*p*-value) to the direction of fold-change. From this list, the significant DEGs were tabulated along with the corresponding ranking metric for use in GSEA. Each list was evaluated for GO enrichment by 1,000 permutations using the default settings in Blast2GO but also to include all data (min = 1). As the entire transcriptome (reference set) was functionally characterized in Blast2GO, Fisher's and GSEA analyses were performed using all significant transcripts (test set) for each comparison. Lastly, each significant DEG list was functionally characterized for GO, enzyme commission number, InterPro assessment and KEGG pathway assignment.

### qPCR Validation

Select genes were independently assessed via reverse transcription quantitative PCR (RT-qPCR) to validate our RNAseq pipeline. For each comparison, all individual DNase-treated RNA used for RNAseq library creation were also tested using qPCR. Each RNA was re-quantified by spectrophotometry (Bio-Tek) and standardized to 5 ng. The iTaq Universal SYBR Green One-Step Kit (Bio-Rad, Hercules, CA) was used for qPCR reactions. Each reaction contained 2 μL RNA, 5 μL of iTaq universal SYBR Green reaction mix (2x), 0.25 μL of iScript reverse transcriptase, 300 nM of each primer and nuclease-free water up to a total 10 μL volume. RT-qPCR was then performed using a Roche LightCycler 96 System (Indianapolis, IN), with cycling parameters according to the iTaq provided instructions. Technical replicates were performed for each reaction. Negative controls and no reverse transcriptase controls were included in each 96-well plate. Each qPCR was assessed by melting curve analysis. After all reactions, cycle threshold (Ct) values were collected and fold-change between each comparison was determined using the 2^−ΔΔCT^ method (48). *P*-values were calculated using a Student's *t*-test. We assessed several sets of published housekeeping gene primer sets (glucuronidase beta, glyceraldehyde-3-phosphate dehydrogenase, alpha tubulin, beta-actin, and 18S ribosomal RNA gene) and found that the 18S ribosomal RNA sets performed the best with skin explant tissue RNA (49–51). Validation primer set sequences are provided (Table S6).

## Results

### Evaluation of Recombinant *Flavobacterium columnare* DnaK Protein

**Figure 1:**
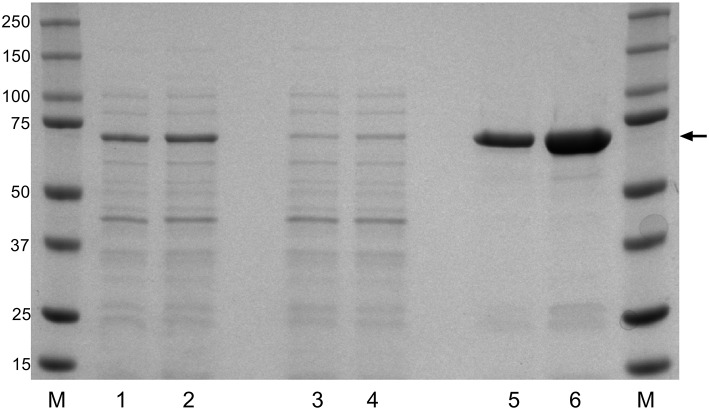
Recombinant expression of *F. columnare* DnaK protein (rDnaK). Coomassie stain of SDS gel of *E. coli* lysates (lanes 1 and 2); Ni-NTA column washes (lanes 3 and 4), and eluted protein fractions (lanes 5 and 6) after 4 or 6 h of IPTG stimulation, respectively. The arrow marks the 70 kDa band representing the approximate size of the eluted rDnaK protein. (M) The pre-stained Precision Plus marker (kDa) was used to estimate molecular mass.

To evaluate the potential of the *F. columnare* DnaK protein as a recombinantly expressed vaccine candidate, the DnaK amino acid sequence was identified among the annotated protein sequences of *F. columnare* isolate ATCC 49512 genome. An alignment of DnaK amino acid sequences from recently released *F. columnare* genomes and protein sequences from other Gram-negative bacteria were compared to that identified in *F. columnare* ATCC 49512 (Table S1). The alignment of the DnaK protein sequences showed an average of 99.3% amino acid sequence identity among the different *F. columnare* isolates with lower amino acid sequence identity (~60%) among the other Gram-negative bacteria. After cloning and expressing the recombinant DnaK protein, Ni-NTA columns were used and provided a highly enriched rDnaK protein which migrated at the expected size (70 kDa). The added time allowed for an increase in protein synthesis between the 4 and 6 h intervals and benefited the overall yield during recombinant expression (Figure 1). The eluted rDnaK protein was later confirmed to be *Flavobacterium columnare* ATCC 49512 DnaK protein (AEW86253.1) through mass spectrometry analysis.

### Efficacy of Immunization Strategies in Year 1

**Figure 2:**
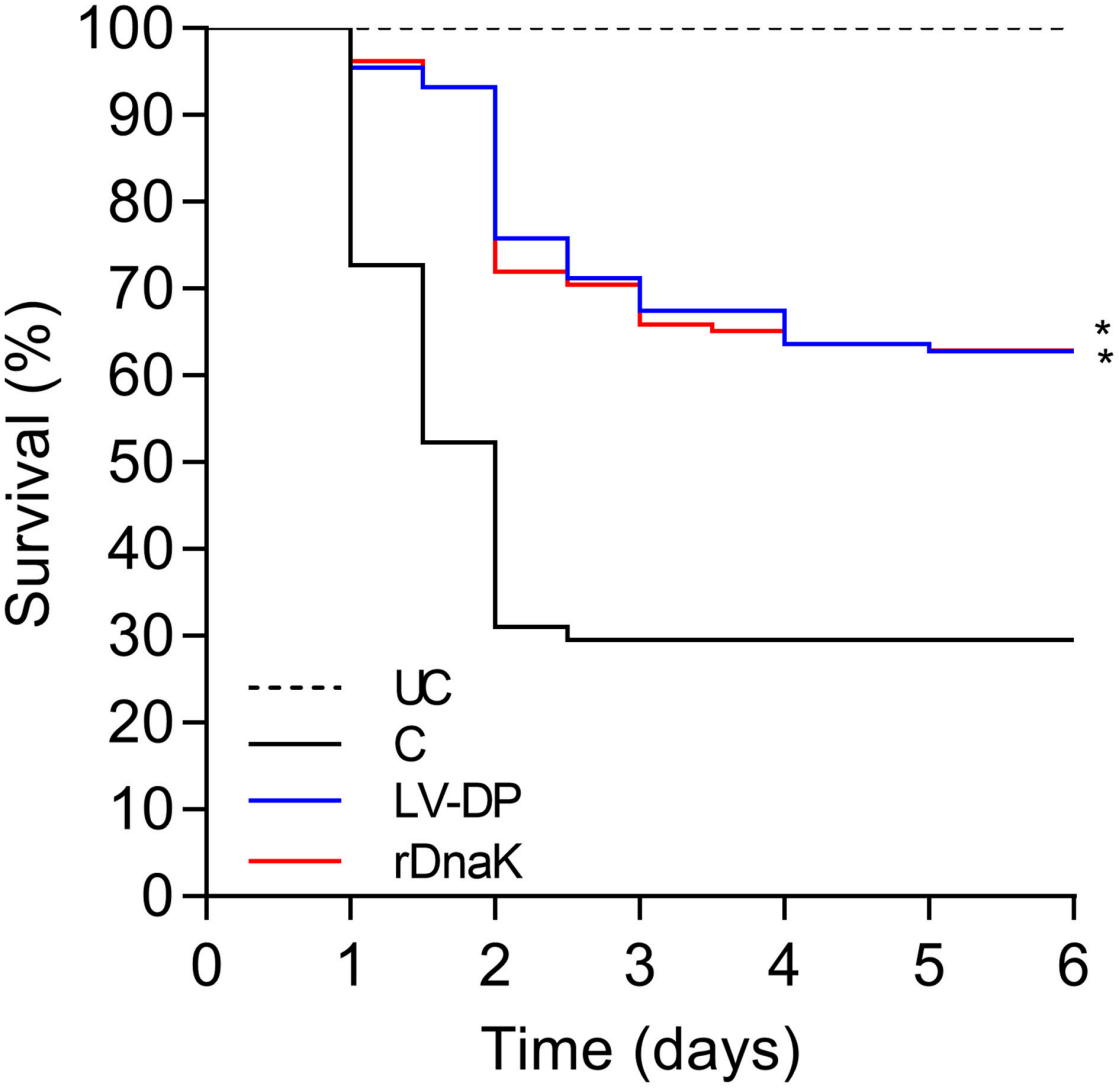
The impact of year-one immunization protocols on the protection of channel catfish against an *F. columnare* laboratory challenge. Kaplan-Meier survival curves of catfish challenged with isolate LV-359-01 over 6 days. The different groups are labeled as indicated in the figure. Data represent cumulative mortality across three replicate tanks per treatment containing 132 fish (*n* = 44/tank). Asterisk(s) denote a significant difference in survival when compared to the non-immunized control group; ^*^P < 0.05.

Six weeks post immunization a laboratory challenge was conducted on the non-immunized control, LV-DP and rDnaK immunized groups with *F. columnare* LV-359-01. Kaplan-Meier survival analysis showed that 6 days post challenge there was a significantly higher survival rate (*P* <0.05) between the two immunized groups when compared to the non-immunized control (Figure 2). There was no significant difference in survival between the immunized groups. The overall survival rate of the immunized groups was 64% while the non-immunized control was 32%. These results indicate that significant protection was achieved in both the LV-DP and rDnaK immunized groups.

### Antibody Response to *F. columnare* Extracellular Proteins in Year 1

**Figure 3:**
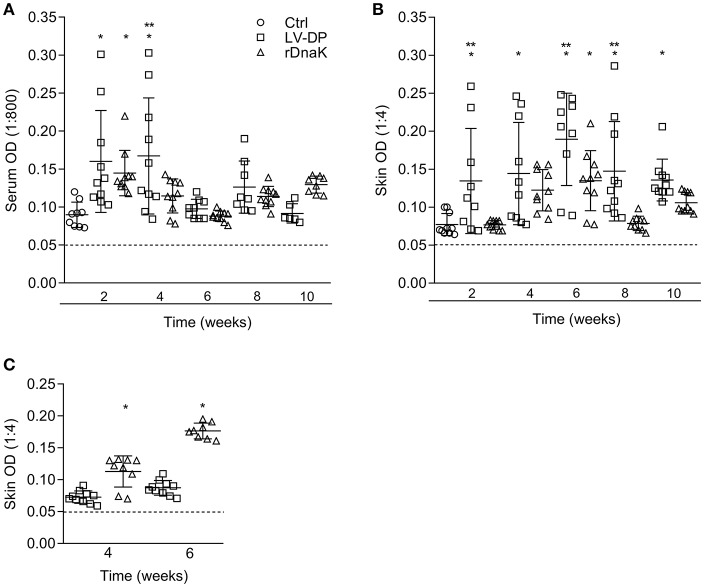
Evaluation of antibodies generated against *F. columnare* ECP and recombinant DnaK protein. The different groups are labeled as indicated in the figure. **(A)** The serum IgM antibody titer (1:800) is the reciprocal of the maximum dilution at which we could reliably detect binding of antibody to *F. columnare* ECP. **(B)** The skin IgM antibody titer (1:4) is the reciprocal of the maximum dilution at which we could reliably detect binding of antibody to *F. columnare* ECP. **(C)** The skin IgM antibody titer (1:4) is the reciprocal of the maximum dilution at which we could reliably detect binding of antibody to *F. columnare* rDnaK. The mean absorbance ± SD for each group is shown as a horizontal line. Asterisk(s) denote a significant difference, *P* <0.05. (^*^), indicates a significant difference when compared to the non-immunized control; (^**^), indicates a significant difference between immunized groups. The dashed line represents the mean background absorbance value observed using an isotype control.

To evaluate the development of antibodies to the *F. columnare* ECP, the serum of non-immunized and immunized catfish collected at different time points was assessed using an indirect ELISA. The absorbance values showed that individual fish had generated varying amounts of serum IgM antibodies to *F. columnare* ECP (Figure 3A). At 2 weeks post immunization the non-immunized control had a mean absorbance value of 0.089 ± 0.01, the LV-DP and rDnaK immunized groups were 0.160 ± 0.06 and 0.145 ± 0.03, respectively. At 4 weeks the LV-DP and rDnaK immunized groups mean absorbance values remained higher than the 2-week control at 0.167 ± 0.07 and 0.115 ± 0.02. There were significantly higher absorbance values (*P* < 0.05) between the two immunized groups at week two and for the LV-DP group at week four. The LV-DP group also had significantly more serum IgM antibodies (*P* < 0.05) that bound to *F. columnare* ECP at week four when compared to the rDnaK group. Over the remaining weeks none of the catfish sampled from either of the two immunized groups generated little more ECP specific serum IgM antibodies than that detected in the non-immunized control. Overall the LV-DP-immunized catfish produced more ECP specific serum antibodies than the rDnaK-immunized fish (with more responsive individuals at weeks four and eight); however, there were no additional significant differences observed between the immunized groups.

Similarly, the humoral immune response of the mucosae was assessed by evaluating the amount of ECP specific IgM antibodies present among the *in vitro* cultured skin explant medium (Figure 3B). At 2 weeks, the non-immunized control had a mean absorbance value of 0.071 ± 0.01, while the LV-DP was 0.134 ± 0.06 and rDnaK was 0.076 ± 0.05. The LV-DP group generated a more significant skin IgM antibody response to ECP (*P* < 0.05) than that observed in the control group at weeks 2, 4, 6, 8, and 10. The rDnaK group also generated more significant skin IgM antibody response to ECP than did the non-immunized control at 6 weeks. Interestingly, the LV-DP group generated significantly more ECP specific mucosal IgM antibodies (*P* < 0.05) than did the rDnaK group at weeks 2, 6, and 8. These results indicate that the bath immunization protocols (LV-DP or rDnaK) stimulated different mucosal humoral immune responses, and overall there was a much more substantial immune response with the LV-DP group.

### Mucosal Antibody Response to Recombinant *F. columnare* DnaK in Year 1

To establish the level at which mucosal IgM antibodies were specifically generated to the *F. columnare* DnaK protein the skin explant tissue culture medium and an rDnaK specific indirect ELISA were used (Figure 3C). Four weeks post immunization the LV-DP immunized group had a mean absorbance of 0.072 ± 0.01 while the rDnaK immunized group was significantly higher at 0.113 ± 0.03 (*P* < 0.05). At 6 weeks LV-DP had generated slightly more rDnaK specific antibodies with a mean absorbance of 0.087 ± 0.01, while the rDnaK group remained significantly higher with a mean absorbance of 0.177 ± 0.01 (*P* < 0.05). These results indicate that both the LV-DP and rDnaK groups stimulated an *F. columnare* DnaK specific mucosal IgM antibody response albeit at much different levels.

### Efficacy of Immunization Strategies in Year 2

**Figure 4:**
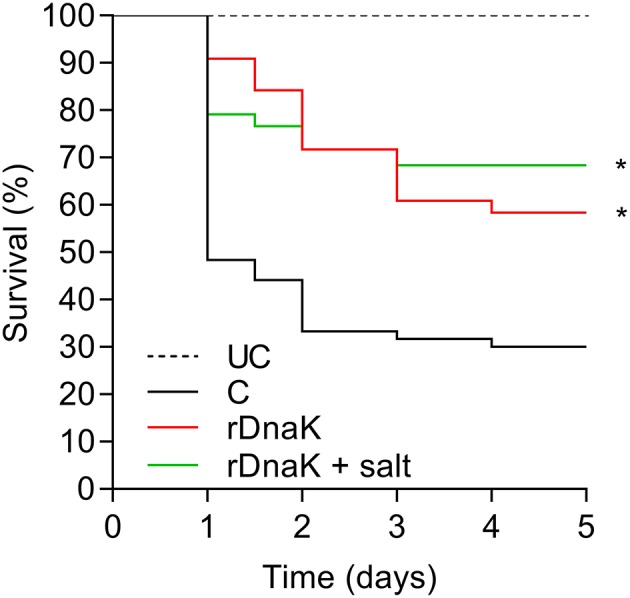
The impact of year-two immunization protocols on the protection of channel catfish against an *F. columnare* laboratory challenge. Kaplan-Meier survival curves of catfish challenged with isolate LV-359-01 over 5 days. The different groups are labeled as indicated in the figure. Data represent cumulative mortality across three replicate tanks per treatment containing 120 fish (*n* = 40/tank). Asterisk(s) denote a significant difference in survival when compared to the non-immunized control group; ^*^P < 0.05.

In the year two trials to test the efficacy of the recombinant *F. columnare* rDnaK protein vaccine there were two immunized groups (rDnaK and rDnaK + salt), the latter received a brief 4.5% NaCl dip prior to bath immunization. The results to the *F. columnare* laboratory challenge indicated there was a significantly higher survival rate (*P* < 0.05) among the immunized groups when compared to the non-immunized control (Figure 4). The overall survival rates of the immunized groups were 57% (rDnaK) and 68% (rDnaK + salt) and 31% survival among the non-immunized group. There was no significant difference in survival between the two rDnaK-immunized groups.

### Antibody Response to Recombinant *F. columnare* DnaK in Year 2

**Figure 5:**
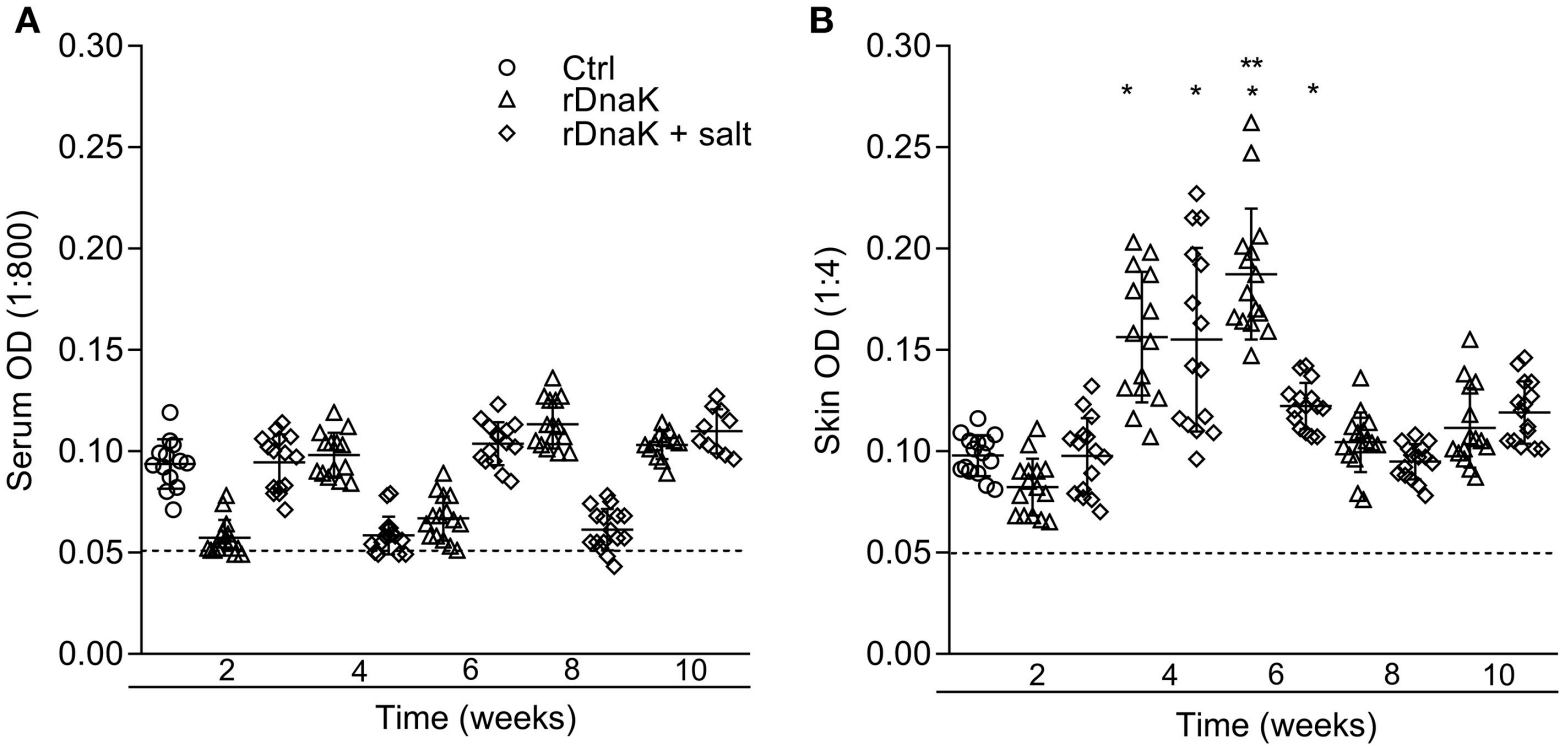
Evaluation of antibodies generated against *F. columnare* recombinant DnaK protein. The different groups are labeled as indicated in the figure. **(A)** The serum IgM antibody titer (1:800) is the reciprocal of the maximum dilution at which we could reliably detect binding of antibody to *F. columnare* rDnaK. **(B)** The skin IgM antibody titer (1:4) is the reciprocal of the maximum dilution at which we could reliably detect binding of antibody to *F. columnare* rDnaK. The mean absorbance ± SD for each group is shown as a horizontal line. Asterisk(s) denote a significant difference, *P* < 0.05. (^*^), indicates a significant difference when compared to the non-immunized control; (^**^), indicates a significant difference between immunized groups. The dashed line represents the mean background absorbance value observed using an isotype control.

To evaluate the development of IgM antibodies to recombinant *F. columnare* DnaK protein, the serum of non-immunized and immunized fish was collected at different time points and evaluated using an rDnaK specific indirect ELISA (Figure 5A). At 2 weeks post immunization the non-immunized control had a mean absorbance value of 0.093 ± 0.01, the (rDnaK) and (rDnaK + salt) immunized groups were 0.057 ± 0.008 and 0.094 ± 0.01, respectively. Over the next several weeks none of the catfish sampled from either of the two immunized groups generated higher absorbance values than that detected in the non-immunized control indicating little to no serum antibodies were generated to the rDnaK protein.

To establish the level at which mucosal IgM antibodies were generated to rDnaK, skin explant tissue culture medium and an rDnaK specific indirect ELISA were used (Figure 5B). At 2 weeks post immunization the non-immunized control had a mean absorbance value of 0.097 ± 0.01, the rDnaK immunized groups were 0.082 ± 0.01 and 0.097 ± 0.01, respectively. The non-immunized control fish never developed rDnaK specific Abs in the skin at the later time points. Both rDnaK immunized groups later generated significantly more skin IgM antibodies to rDnaK (*P* < 0.05) than that observed in the non-immunized group at weeks four and six. The rDnaK group also generated significantly more *F. columnare* rDnaK antibodies (*P* < 0.05) than the (rDnaK + salt) group at 6 weeks. These results indicate both immunized groups stimulated *F. columnare* rDnaK specific mucosal IgM antibody responses.

### Differential Gene Expression and Gene Ontology of Skin Explants in Year 2

There was a total of six RNA sequence libraries prepared and sequenced from the skin of individual catfish from the non-immunized control and rDnaK groups (week one and six) for a total of 18 RNAseq libraries. Following the processing of the raw RNA sequencing data and alignment with the *I. punctatus* transcriptome, we conducted comparisons between the control and treatment groups at different time intervals to establish their gene expression profiles. In each case, the first biological state listed is the base (reference) level. Three comparisons of interest were made including: non-immunized control and rDnaK immunized group (week one), non-immunized control and rDnaK immunized group (week six), and rDnaK (week one) and rDnaK (week six). For each of the three comparisons we identified between 1.0 and 5.5% of the total genes identified among the different transcriptomes as being differentially expressed according to the following cutoff criteria (*P*-adj <0.05, Fold change >1.5). There were 420 differentially expressed genes (DEGs) between the non-immunized control and rDnaK group 1-week post immunization that included 323 upregulated and 96 down regulated genes (Data Set 1). There were 579 DEGs between the non-immunized control and the rDnaK group (week six), including 472 upregulated and 106 downregulated genes (Data Set 2). There were 52 DEGs identified between the rDnaK groups (week one and six) with 17 upregulated and 35 down regulated genes (Data Set 3).

Gene ontology analyses were performed on the DEGs from each comparison to determine functional enrichment. Among the non-immunized control and rDnaK groups at 1-week post immunization, there were 157 over-represented GO groups: 123 BP, 28 MF, and 6 CC categories. The top five most significant GO names identified in each category are represented by processes such as carbohydrate biosynthesis (GO: 0016051), oxireductase activity (GO: 0016491) and intermediate filament (GO: 0005882) (Table S2). The same analysis was conducted between the non-immunized control and the rDnaK group (week 6) resulting in 180 over-represented GO groups: 143 BP, 28 MF, and 9 CC categories (Table S3). The top five most significant GO names identified in each category are represented by processes such as ATP generation (GO: 0006757), endopeptidase inhibitor activity (GO: 0004866), and intermediate filament (GO: 0005882). There were no significantly represented GO groups identified among the DEGs of the rDnaK groups (week one and six).

### Identification of Genes Associated With Immune Function in Skin Explants

To evaluate the DEGs that may be involved with skin immune function a GO distribution level 2 analysis was conducted with the top 20 GO terms represented by the non-immunized control and rDnaK group at week one (Figure S1). Through this analysis we identified 17 DEGs associated with the GO term immune system process (GO: 0002376) (Table S4). Further testing for the representation of enzymes in KEGG pathways identified three genes involved in T-cell differentiation and receptor signaling pathways (maps 04658 and 04660). The GSEA also identified GO terms associated with myeloid leukocyte activation (GO: 0002274) and immune effector process (GO: 00052252).

The same evaluations were then conducted between the non-immunized control and the rDnaK group at week six (Figure S2). The DEGs identified 23 genes associated with immune system processes (GO: 0002376) at GO level 2, and seven genes involved in T-cell differentiation and receptor signaling pathways (maps 04658 and 04660) through KEGG analysis (Table S5). GSEA identified additional GO terms involved with immunity, including myeloid leukocyte activation (GO: 0002274), regulation of immune effector process (GO: 0002697), cell chemotaxis (GO: 0060326) and leukocyte mediated immunity (GO: 0002443).

### Validation of RNA Sequencing

Reverse transcription qPCR was conducted on skin explant RNA samples submitted for library preparation and sequencing. We chose four genes that were both up- and down-regulated among the rDnaK immunized group at different time points for validation of our RNA sequencing analysis (Table S6). In all qPCR experiments, average 18S Ct values were within <1 Ct unit of each other among the different RNA samples (Table S7). All qPCR data was statistically significant and correlated to the RNA sequencing data (Table S8).

## Discussion

Columnaris disease continues to be a significant issue among the different production systems of warmwater finfish. *F. columnare* infections principally begin as biofilms on the different mucosal surfaces causing significant damage to the gills and skin (52–54). To avert disease and reduce losses the current study sought to evaluate the potential of the recombinant *F. columnare* DnaK protein to activate a mucosal IgM antibody response in channel catfish and assess the level of protection which could be generated during an *F. columnare* challenge. We were also interested in a time-course assessment of a mucosal tissue to determine potential functional mechanisms involved in this protective immunity.

The use of recombinant protein technology to develop new vaccines is not a new concept and is widely being examined for use in both human and animal health (55–58). Our rationale for choosing this protein was due to a previous study in which DnaK was shown to be the dominant immunogen in the *F. columnare* ECP. The *F. columnare* chaperone protein DnaK was recombinantly expressed, purified and used as a soluble antigen in a bath immersion vaccine. We and others have demonstrated that fish can generate IgM antibodies that are immune-reactive to DnaK proteins found among different Gram-negative bacteria (26, 59). DnaK proteins have also been demonstrated to be highly immunogenic (29, 31, 60). An alignment of DnaK protein sequences among different *F. columnare* genomes indicated a high percentage of amino acid similarity, which led us to hypothesize that the rDnaK protein may induce cross protection between different *F. columnare* isolates.

One of the most important parameters evaluated during vaccine efficacy studies is the ability for the immunogen(s) to confer immune protection against the known pathogen. Previous work in rainbow trout indicated that *F. psychrophilum* heat shock proteins (similar to DnaK chaperone protein) were highly immunogenic but not protective against bacterial cold-water disease (59, 61). The opposite was observed in the current study where 2 years of rDnaK vaccine trials demonstrated that significant protection is achieved after bath immunization. From year-to-year and among the different immunization strategies, we observed very similar increases in survival (~30%). More importantly in year one we demonstrated that the rDnaK protein can confer protection at a similar rate to live-attenuated bacterial cells and ECP. These results suggest that rDnaK may be a valuable vaccine candidate in providing protection against columnaris disease. We are also confident that the rDnaK immunogen can be further optimized using improved vaccination strategies. Unlike other vaccine trials in fish, the immunization strategies used in this study were performed independent of any conventional adjuvants (62–64). In year two, the use of hyperosmotic induction prior to bath immunization increased overall survival and has been shown by others to have a positive effect on soluble antigen uptake and mucosal antibody production (65). The use of a commercial mucosal adjuvant in tandem with an osmotic shock pre-treatment could prove to be a valued bath immersion vaccine strategy (66, 67).

To evaluate the adaptive immune response, IgM antibodies produced at different time intervals were assessed using a rDnaK-specific ELISA. Similar to what we and others have observed, the use of a soluble antigen to stimulate the external mucosae has generated mucosal IgM antibodies (20, 22, 68). During both vaccine trials rDnaK-specific antibodies were most abundant in the skin during weeks four and six, however all rDnaK-specific antibodies fell to baseline levels by weeks 8 and 10 in the skin of both immunized groups. The overall result is consistent with what we have previously observed (22). Among the later time points it is possible that the limits of detection for the rDnaK-specific ELISA have been reached. As this assay relies on mucosal antibody secretion into the tissue culture medium, a minimum number of antibody-secreting cells would likely need to be present. Perhaps for long term analysis another assay should be performed. In fact others have established through the use of an ELISPOT assay that antibody-secreting cells could be enumerated from lymphocytes isolated from the skin (69). This work showed that the number of antibody-secreting cells remained elevated in the skin for up to 17 weeks after immunization. In later studies, the same group would demonstrate that despite the loss of circulating mucosal antibodies; antibody-secreting cells that were present in the skin were activated after a significant amount of time and that immune protection was retained (70). Perhaps IgM memory B cells are generated during the bath immunization with rDnaK protein. Clearly the amount of antigen(s), the duration of exposure and antigen complexity will govern the magnitude of antibody responses to individual proteins. Additional testing will need to be performed to determine the longevity of protection among the rDnaK-immunized groups (71).

Analyzing gene expression in the skin of the non-immunized and immunized groups at different time points, we were able to identify DEGs and characterize functionally enriched processes associated with different biosynthetic and transport pathways. The most interesting observation was among the cellular component groups of the rDnaK skin (weeks one and six) which showed an overrepresentation of intermediate filament, intermediate filament cytoskeleton and desmosome (GO groups). The desmosomes are characterized as adhesive intercellular junctions which facilitate skin integrity between individual cells (72). The fish skin is the host's primary barrier to infection and is the frontline defense against infection (73). The integument is vital in its capacity to maintain both structure and regulate the mucosal environment from both friend and foe (74). Li et al. characterized the up- and downregulation of different genes in catfish skin associated with junctional/adhesion groups after an *Aeromonas hydrophila* infection (75). Perhaps during an active mucosal immune response in the skin, the mechanisms which regulate skin integrity and cell infiltration are activated in addition to immune-related genes to ensure that homeostasis is maintained (76).

We also identified candidate genes associated with immune function, and while we don't necessarily have a complete picture of what is occurring in the immunized skin we can make some general observations from our findings. There were molecular processes associated both with immune effector function and regulation of immune effector (rDnaK week one and six) which identified the CD59 glycoprotein-like and matrix metalloproteinase 9 genes, each of which could have a role in innate immune function (77, 78). There were also processes characterized as cell chemotaxis (week six) which identified sphingosine 1-phopshate receptor 4 like, ras-related C3 botulinum toxin substrate 2 and CxC motif chemokine 2-like genes underlying this functional group. Interestingly the cell chemotaxis GO term wasn't enriched at week one, however the sphingosine 1-phopshate receptor 4 like, ras-related C3 botulinum toxin substrate 2 genes were also upregulated. The sphingosine phosphate family of receptors has been implicated in both B and T cell migration in mammals (79, 80).

Lastly, it is evident that B cells were activated and differentiated into rDnaK-specific antibody secreting cells in the skin during the 4 to 6-week interval. A few genes associated with T cell signaling and differentiation (tyrosine-protein kinase Lck-like, protein tyrosine phosphatase, non-receptor type 13, dual specificity phosphatase 14, tyrosine-protein phosphatase non-receptor type 3-like, and 14 kDa phosphohistidine phosphatase-like) were identified in one or both time points and could signify the coordination of T cells in the skin during an adaptive immune response. In general, there is limited information on the fish mucosal T cell response, although different subsets of T cells are likely present in these tissues (66, 81).

The fundamental role of developing a skin cell-mediated immune response and its contribution to overall immune protection against different fish pathogens will need to be further explored. We will continue to investigate the adaptive immune response in fish using conventional immunological assays as well as using genomics technologies to determine optimal conditions for long lasting immunization procedures.

## Ethics Statement

Animal care and experimental protocols were approved by the Stuttgart National Aquaculture Research Center Institutional Animal Care and Use Committee and conformed to USDA Agricultural Research Service Policies and Procedures 130.4 and 635.1.

## Author Contributions

ML and JA designed the experiments, analyzed the data, and prepared the figures, and drafted the manuscript. ML and BF performed vaccination and challenge studies. JA prepared RNA sequencing libraries and performed the related bioinformatics. All authors contributed to the final manuscript.

### Conflict of Interest Statement

The authors declare that the research was conducted in the absence of any commercial or financial relationships that could be construed as a potential conflict of interest.
