# Supplementary material for: Evaluation of a Recombinant Flavobacterium columnare DnaK Protein Vaccine as a Means of Protection Against Columnaris Disease in Channel Catfish (Ictalurus punctatus)
Source: Front Immunol. 2019 Jun 6;10:1175. doi: 10.3389/fimmu.2019.01175 (PMC6562308; doi:10.3389/fimmu.2019.01175)
Supplement: Supplementary file 4 [file Data_Sheet_1.docx]

***Supplementary Material***

**Evaluation of a recombinant *Flavobacterium columnare* DnaK protein vaccine as a means of protection against columnaris disease in channel catfish (*Ictalurus punctatus*)**

**Miles D. Lange, Jason Abernathy and Bradley D. Farmer**

**Correspondence:** Miles D. Lange: miles.lange@ars.usda.gov

1. Supplementary Data

Data Set 1. Gene Expression Between Non-Immunized Control and rDnaK Week 1 Skin Explant Samples.

Data Set 2. Gene Expression Between Non-Immunized Control and rDnaK Week 6 Skin Explant Samples.

Data Set 3. Gene Expression Between rDnaK Week 1 and rDnaK Week 6 Skin Explant Samples.

1. Supplementary Figures and Tables

Figures S1 and S2

Tables S1-S8


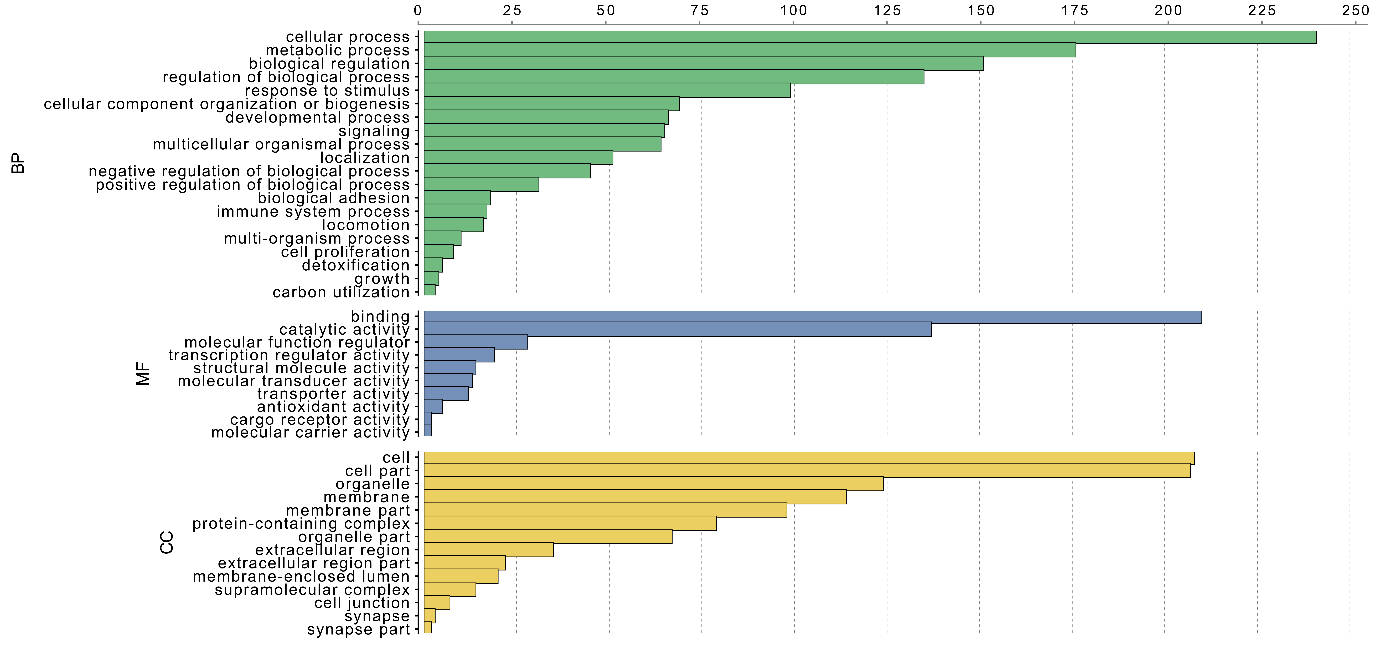


Figure S1. GO Distribution for Level 2 Between Non-immunized Control Week 1 and rDnaK Week1 Skin Explant Samples. BP: Biological Process, MF: Molecular Function, CC: Cellular Component. The x-axis indicates the number of sequences identified in each GO term.


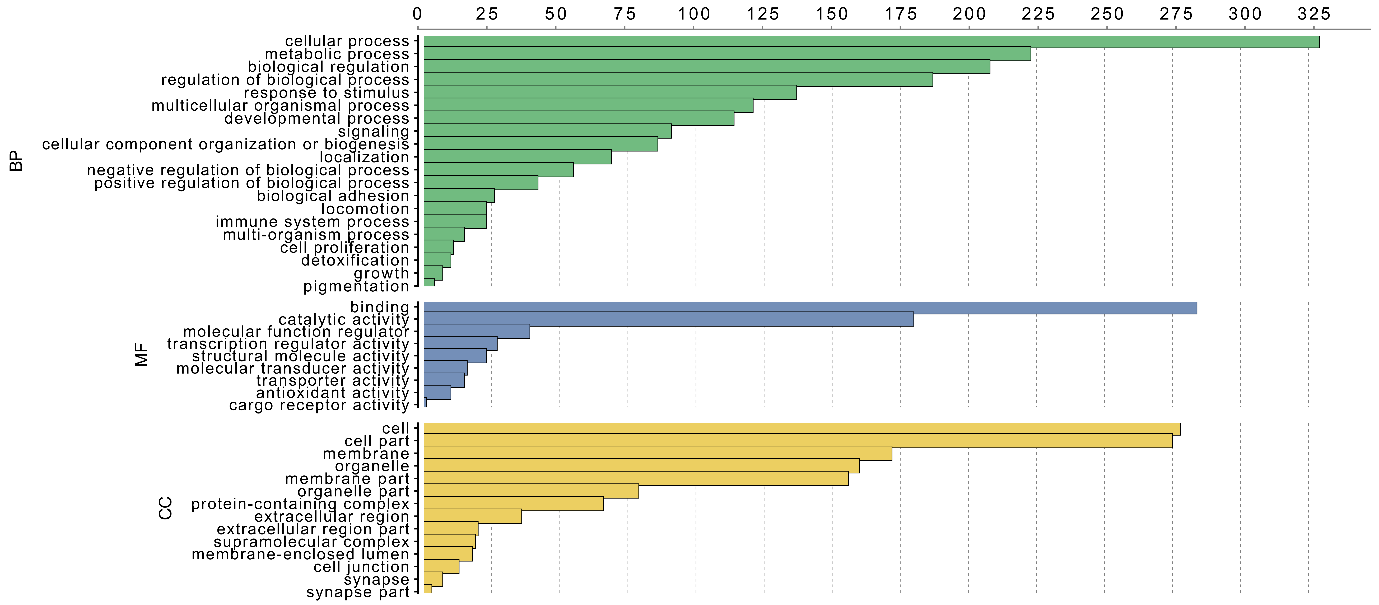


Figure S2. GO Distribution Level 2 Between Non-immunized Control Week 1 and rDnaK Week 6 Skin Explant Samples. BP: Biological Process, MF: Molecular Function, CC: Cellular Component. The x-axis indicates the number of sequences identified in each GO term.

Table S1. Comparison of DnaK amino acid sequences from *Flavobacterium columnare* isolates and other Gram-negative bacteria.

| Bacterial Isolates | Protein  Accession  Number^a^ | Amino Acid Identity (%) to AEW86253^b^ |
| --- | --- | --- |
| *Flavobacterium columnare* Pf1  *Flavobacterium columnare* 1691  *Flavobacterium columnare*  C#2  *Flavobacterium columnare* 94-081  *Flavobacterium johnsoniae* UW 101  *Flavobacterium psychrophilum* JIP02/86  *Borrelia burgdorferi* B31  *Chlamydia trachomatis*  *Pseudomonas aeruginosa* PAO1  *Aeromonas hydrophila* | ANO48542  APT23406  AND65310  AMA48564  A5FGL1  A6GXZ1  NP_212652  WP_009872626  Q9HV43  KLV44233 | 99.8  99.8  98.8  98.8  81.6  80.3  62.7  60.2  59.2  58.7 |

^a^ Protein accession numbers correspond to the chaperone protein DnaK identified among the coding sequences of different *Flavobacterium* sp. and Gram-negative bacteria.

^b^ DnaK amino acid sequence annotated from the *F. columnare* ATCC 49512 genome (CP003222).

Table S2. Gene ontology enrichment in skin explant samples one week after bath immunization with rDnaK. The top five most significant overrepresented gene ontology names in each category are shown.

| GO Category^a^ | GO ID | GO Name | FDR^b^ |
| --- | --- | --- | --- |
| BP | GO:0016051 | Carbohydrate biosynthetic process | 1.91E-07 |
|  | GO:0006757 | ATP generation from ADP | 1.91E-07 |
|  | GO:0042866 | Pyruvate biosynthetic process | 1.91E-07 |
|  | GO:0006165 | Nucleoside diphosphate phosphorylation | 1.91E-07 |
|  | GO:0046939 | Nucleotide phosphorylation | 1.91E-07 |
| MF | GO:0016491 | Oxidoreductase activity | 2.9E-05 |
|  | GO:0015129 | Lactate transmembrane transporter activity | 0.000551 |
|  | GO:0019200 | Carbohydrate kinase activity | 0.001328 |
|  | GO:0008443 | Phosphofructokinase activity | 0.001548 |
|  | GO:0005507 | Copper ion binding | 0.001863 |
| CC | GO:0005882 | Intermediate filament | 1.26E-05 |
|  | GO:0045111 | Intermediate filament cytoskeleton | 1.52E-05 |
|  | GO:0030057 | Desmosome | 0.002522 |
|  | GO:0005581 | Collagen trimer | 0.007977 |
|  | GO:0005576 | Extracellular region | 0.010294 |

^a^ BP: Biological Process, MF: Molecular Function, CC: Cellular Component.

^b^ The differentially expressed genes were evaluated for enrichment using Fisher’s exact test at a False Discovery Rate (FDR < 0.05).

| GO Category^a^ | | GO ID | GO Name | FDR^b^ |
| --- | --- | --- | --- | --- |
| BP | GO:0006757 | ATP generation from ADP | 5.46E-08 |  |
|  | GO:0042866 | Pyruvate biosynthetic process | 5.46E-08 |  |
|  | GO:0009166 | Nucleotide catabolic process | 5.46E-08 |  |
|  | GO:0006096 | Glycolytic process | 5.46E-08 |  |
|  | GO:0016051 | Carbohydrate biosynthetic process | 6.47E-08 |  |
| MF | GO:0004866 | Endopeptidase inhibitor activity | 1.22E-06 |  |
|  | GO:0016491 | Oxidoreductase activity | 1.60E-06 |  |
|  | GO:0061135 | Endopeptidase regulator activity | 1.86E-06 |  |
|  | GO:0030414 | Peptidase inhibitor activity | 3.13E-06 |  |
|  | GO:0004601 | Peroxidase activity | 3.13E-06 |  |
| CC | GO:0005882 | Intermediate filament | 6.47E-08 |  |
|  | GO:0045111 | Intermediate filament cytoskeleton | 7.79E-08 |  |
|  | GO:0030057 | Desmosome | 8.21E-06 |  |
|  | GO:0098636 | Protein complex involved in cell adhesion | 5.22E-06 |  |
|  | GO:0008305 | Integrin complex | 5.22E-06 |  |

Table S3. Gene ontology enrichment in skin explant samples six weeks after bath immunization with rDnaK. The top five most significant overrepresented gene ontology names in each category are shown.

^a^ BP: Biological Process, MF: Molecular Function, CC: Cellular Component.

^b^ The differentially expressed genes were evaluated for enrichment using Fisher’s exact test at a False Discovery Rate (FDR < 0.05).

Table S4. Candidate list of genes associated with immune function in skin explant samples one week after bath immunization with rDnaK.

| Gene ID | Description | Fold change | Significance |
| --- | --- | --- | --- |
| XM_017473160.1 | Transcriptional regulator Myc-B-like (LOC108268289) | -3.21 | DEG |
| XM_017456779.1 | Toll-like receptor 1 (LOC108258270) | -2.22 | DEG |
| XM_017477521.1 | Syntaxin 6 (stx6), transcript variant X1 | -1.90 | DEG |
| XM_017456213.1 | Heat shock protein 90 alpha family class B member 1 (hsp90ab1) | 1.76 | DEG |
| XM_017481866.1 | Atypical chemokine receptor 3 (ackr3) | 2.53 | DEG |
| XM_017474751.1 | Secreted protein acidic and cysteine rich (sparc) | 2.78 | DEG |
| XM_017491066.1 | SUMO1 activating enzyme subunit 1 (sae1) | 2.89 | DEG |
| XM_017477458.1 | Ectonucleotide pyrophosphatase/phosphodiesterase 2 (enpp2) | 3.71 | DEG |
| XM_017489163.1 | Endoplasmic reticulum aminopeptidase 1-like (LOC108276965) | 4.04 | DEG |
| XM_017496387.1 | CD59 glycoprotein-like (LOC108280891) | 4.58 | DEG, GSEA |
| XM_017477863.1 | Sphingosine 1-phosphate receptor 4-like (LOC108270882) | 5.20 | DEG |
| XM_017466807.1 | Matrix metalloproteinase 9 (mmp9) | 5.94 | DEG, GSEA |
| XM_017464962.1 | Uncharacterized LOC108263802 (LOC108263802) | 6.66 | DEG, GSEA |
| XM_017488115.1 | Ras-related C3 botulinum toxin substrate 2 (LOC108276437) | 6.70 | DEG, GSEA |
| XM_017476623.1 | Tyrosine-protein kinase Lck-like (LOC108270195) | 7.25 | DEG |
| XM_017454757.1 | Protein NDRG1-like (LOC108257201) | 9.40 | DEG, GSEA |
| XM_017492308.1 | Protein tyrosine phosphatase, non-receptor type 13 (ptpn13) | 10.84 | DEG |
| XM_017477925.1 | Ring finger and CCCH-type domains 1 (rc3h1) | 12.23 | DEG |
| XM_017490121.1 | Dual specificity phosphatase 14 (dusp14) | 16.70 | DEG |
| XM_017474695.1 | Regakine-1-like (LOC108269115) | 45.08 | DEG |

Table S5. Candidate list of genes associated with immune function in skin explant samples six weeks after bath immunization with rDnaK.

| Gene ID | Description | Fold change | Significance |
| --- | --- | --- | --- |
| XM_017490305.1 | Translocase of inner mitochondrial membrane 50 (timm50) | -7.25 | DEG |
| XM_017456794.1 | Dpy-30, histone methyltransferase complex regulatory subunit (dpy30) | -2.74 | DEG |
| XM_017472168.1 | Tyrosine kinase non-receptor 1 (tnk1) | -2.44 | DEG |
| XM_017473160.1 | Transcriptional regulator Myc-B-like (LOC108268289) | -2.18 | DEG |
| XM_017477521.1 | Syntaxin 6 (stx6) | -1.83 | DEG |
| XM_017456213.1 | Heat shock protein 90 alpha family class B member 1 (hsp90ab1) | 1.78 | DEG |
| XM_017496349.1 | SBDS ribosome assembly guanine nucleotide exchange factor (sbds) | 2.44 | DEG |
| XM_017453178.1 | Tyrosine-protein phosphatase non-receptor type 3-like (LOC108256378) | 3.01 | DEG |
| XM_017489163.1 | Endoplasmic reticulum aminopeptidase 1-like (LOC108276965) | 5.24 | DEG |
| XM_017456746.1 | NF-kappa-B inhibitor alpha-like (LOC108258222) | 5.29 | DEG |
| XM_017476549.1 | Runt related transcription factor 2 (runx2) | 6.22 | DEG |
| XM_017469379.1 | Splicing factor 3b subunit 1 (sf3b1) | 6.24 | DEG |
| XM_017457386.1 | Interferon-induced GTP-binding protein Mx2-like (LOC108258627) | 6.25 | DEG |
| XM_017466807.1 | Matrix metalloproteinase 9 (mmp9) | 6.62 | DEG, GSEA |
| XM_017460851.1 | Spermine oxidase (smox) | 6.66 | DEG |
| XM_017475576.1 | Serine peptidase inhibitor, Kunitz type 1 (spint1) | 7.34 | DEG |
| XM_017464962.1 | Uncharacterized LOC108263802 (LOC108263802) | 7.52 | DEG |
| XM_017454757.1 | Protein NDRG1-like (LOC108257201) | 8.10 | DEG, GSEA |
| XM_017477863.1 | Sphingosine 1-phosphate receptor 4-like (LOC108270882) | 10.25 | DEG, GSEA |
| XM_017495153.1 | Transient receptor potential cation channel subfamily M member 4-like (LOC108280300) | 10.31 | DEG, GSEA |
| XM_017488115.1 | Ras-related C3 botulinum toxin substrate 2 (LOC108276437) | 11.15 | DEG, GSEA |
| XM_017457388.1 | Interferon-induced GTP-binding protein Mx2-like (LOC108258629) | 12.11 | DEG |
| XM_017467755.1 | H-2 class II histocompatibility antigen, I-E beta chain-like (LOC108265442) | 12.98 | DEG |
| XM_017476623.1 | Tyrosine-protein kinase Lck-like (LOC108270195) | 14.14 | DEG |
| XM_017450727.1 | Proto-oncogene tyrosine-protein kinase Src-like (LOC108255075) | 14.86 | DEG |
| XM_017476519.1 | Protein-tyrosine kinase 2-beta-like (LOC108270138) | 19.90 | DEG |
| XM_017455376.1 | 14 kDa phosphohistidine phosphatase-like (LOC108257519) | 19.93 | DEG |
| XM_017476519.1 | Protein-tyrosine kinase 2-beta-like (LOC108270138) | 19.93 | DEG |
| XM_017451502.1 | Tet methylcytosine dioxygenase 3 (tet3) | 28.06 | DEG |
| XM_017496039.1 | Interleukin-20 receptor subunit beta-like (LOC108280709) | 42.92 | DEG |
| XM_017461117.1 | C-X-C motif chemokine 2-like (LOC108260679) | 75.99 | DEG, GSEA |

Table S6. Primers used for qPCR validation.

| Description | Forward | Reverse | Reference |
| --- | --- | --- | --- |
| Keratin | GGACGCTACTCCATGAAACT | TCCTGACCTTGCCTCTCTAT | This study |
| E3 ubiquitin-protein ligase | CACAACCGGCCAGTCTAAA | CCGACTCGGTCCTTTCATTT |  |
| Aflatoxin B1 aldehyde reductase | AGCAGCTTCAGGAGAACTTG | GACACTCGTGAGCCACTAAAT |  |
| Selenoprotein | CGTCATCGTCGTCGTCATAAA | CCAGAATTCAGTGCAGGAAATAAG |  |
| 18S ribosomal RNA | GAGAAACGGCTACCACATCC | GATACGCTCATTCCGATTACAG | [1] |

Table S7. Average Ct values derived from qPCR amplification of the 18S ribosomal RNA housekeeping gene.

| qPCR reaction | 18S Average Ct values^a^ | | |
| --- | --- | --- | --- |
|  | Control week 1 | rDnaK week 1 | rDnaK week 6 |
| Keratin | 14.29 | 13.75 | 13.79 |
| E3 ubiquitin-protein ligase | 13.17/13.54^b^ | 12.81 | 14.04 |
| Aflatoxin | 12.47 | 11.77 | ND^b^ |
| Selenoprotein | 12.54 | ND^b^ | 12.13 |

^a^ The average Ct values were generated from six separate qPCR reactions using skin explant RNA.

^b^ The average Ct values represent two separate control week 1 amplifications associated with either rDnaK week 1 or week 6.

^c^ Not determined.

| Treatment | Reference | Gene ID | Description | Fold change | |
| --- | --- | --- | --- | --- | --- |
|  |  |  |  | RNA Seq | qPCR |
| rDnaK week 1 | Ctrl. week 1 | XM_017454447.1 | Keratin, type I cytoskeletal 50 kDa-like (LOC108257047) | 22.6 | 29.9 |
| week 6 |  |  |  | 15.3 | 16.3 |
| rDnaK week 1 |  | XM_017473267.1 | E3 ubiquitin-protein ligase RNF182-like (LOC108268348) | 14.3 | 11.9 |
| week 6 |  |  |  | 34.1 | 33.1 |
| rDnaK week 1 |  | XM_017450965.1 | Aflatoxin B1 aldehyde reductase member 4-like (LOC108255199) | -4.0 | -1.8 |
| week 6 |  | XM_017475750.1 | Selenoprotein N, 1 (sepn1) | -6.9 | -1.5 |

Table S8. Quantitative PCR validation of RNA sequencing of catfish skin explant samples from the non-immunized control and rDnaK groups at different time points.

**References**

[1] D. Zhang, W. Thongda, C. Li, H. Zhao, B.H. Beck, H. Mohammed, C.R. Arias, and E. Peatman, More than just antibodies: Protective mechanisms of a mucosal vaccine against fish pathogen Flavobacterium columnare. Fish Shellfish Immunol 71 (2017) 160-170.
